# Spontaneous Retroperitoneal Bleed Coincided With Massive Acute Deep Vein Thrombosis as Initial Presentation of COVID-19

**DOI:** 10.7759/cureus.9772

**Published:** 2020-08-15

**Authors:** Burak Erdinc, Jilmil S Raina

**Affiliations:** 1 Internal Medicine, Brookdale University Hospital Medical Center, New York City, USA

**Keywords:** covid-19, sars-cov-2, retroperitoneal bleeding, acute deep vein thrombosis, hypercoagulable state, compartment syndrome

## Abstract

Severe acute respiratory syndrome coronavirus 2 (SARS-CoV-2) is a novel coronavirus that has caused a global pandemic and unfortunately has caused a health crisis. When severe, coronavirus disease 2019 (COVID-19) can manifest with bilateral pneumonia and hypoxemic respiratory failure but also can affect different organ systems. SARS-CoV-2 infection is known to cause a hypercoagulable state resulting in acute thrombotic events, including venous thromboembolism, acute myocardial infarction, acute stroke, acute limb ischemia, and clotting of ECMO (extracorporeal membrane oxygenation) and CRRT (continuous renal replacement therapy) catheters. Even though it commonly causes thrombotic complications, bleeding complications of COVID-19 due to coagulopathy and use of anticoagulation are less commonly reported. We herein present a case of a patient with COVID-19 complicated by spontaneous retroperitoneal bleeding and massive deep vein thrombosis (DVT), which was later complicated by compartment syndrome. To the best of our knowledge, coexistence of spontaneous bleeding with massive DVT has not been reported in the current literature. This case emphasizes that COVID-19 induced hypercoagulable state can cause massive thrombosis, and patients might need anticoagulation therapy. However, clinicians should also consider the risk of hemorrhagic complications of the disease and be cautious when administering anticoagulant therapy in selected cases.

## Introduction

A recent outbreak of novel coronavirus, severe acute respiratory syndrome coronavirus-2 (SARS-CoV-2), leading to severe pneumonia emerged in Wuhan, Hubei Province, China. SARS-CoV-2 is an enveloped, non-segmented positive-sense RNA virus belonging to the Beta-Coronaviridae family [1]. According to the World Health Organization (WHO), there are more than 16 million confirmed cases and 650,000 deaths from COVID-19 globally. The most common presenting symptoms are fever, cough, fatigue, sore throat, diarrhea, headache, and loss of taste or smell. COVID-19 may cause a severe inflammatory state leading to the disorder of hemostasis. It is also linked with various coagulation abnormalities, such as an increase in procoagulant factor levels, including fibrinogen, D-dimers, and factor VIII that have been associated with higher mortality and increased risk of venous thromboembolism (VTE). This combination of inflammation and thrombosis can be referred to as thromboinflammation or COVID-19 associated coagulopathy. Sepsis-induced coagulopathy and disseminated intravascular coagulopathy (DIC) have been reported with severe disease, mainly in non-survivors. However, most patients with severe infection of SARS-CoV-2 develop thrombosis rather than bleeding, while bleeding is commonly seen in DIC. Severe COVID-19 increases fibrinogen levels and factor VIII activity which is not seen in patients with DIC [2,3]. Here we present a case of COVID-19 infection that reports both thrombosis and bleeding.

## Case presentation

**Figure 1:**
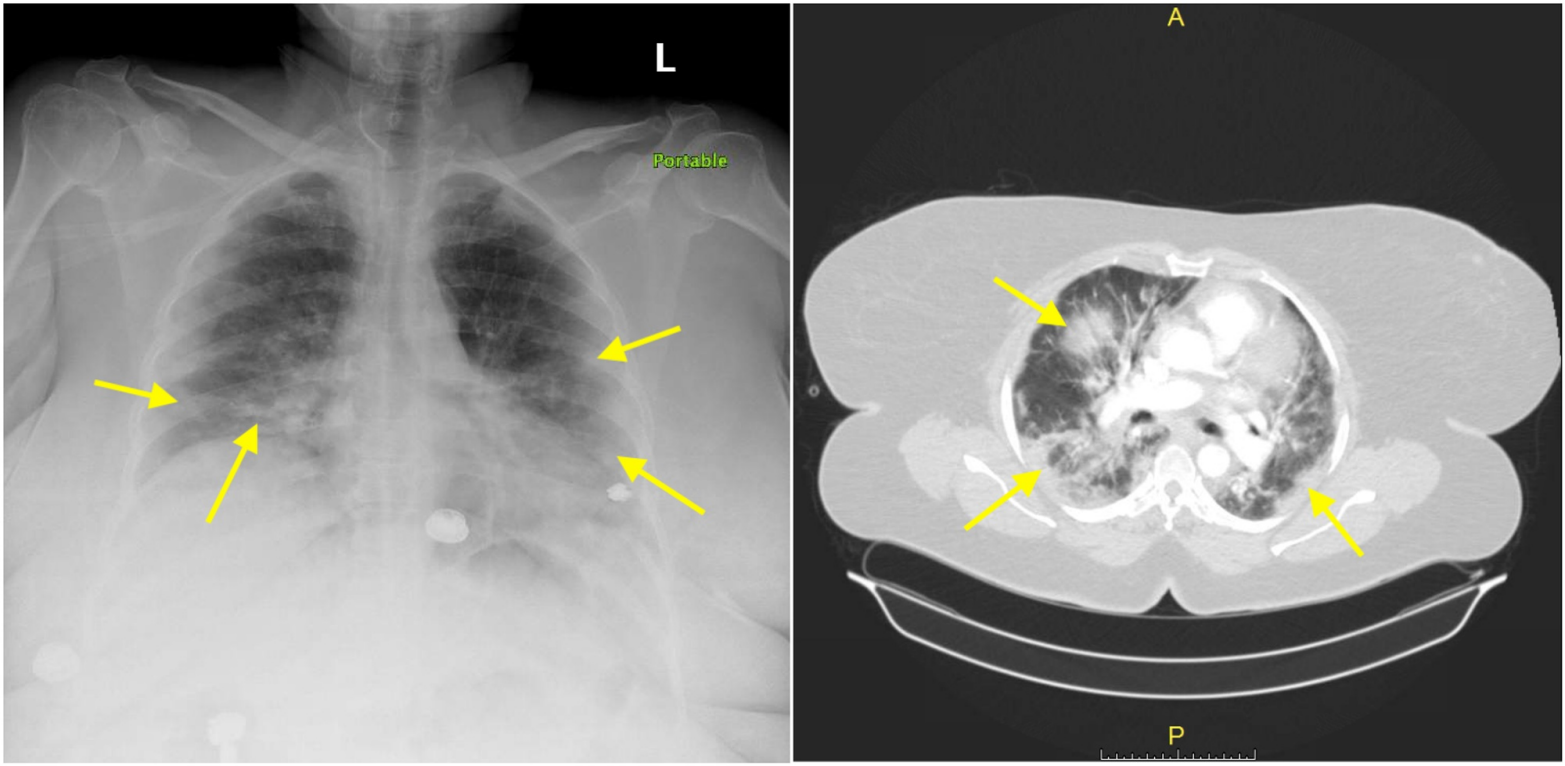
Chest X-ray (on left) and CT angiogram of the chest (on right) showing bilateral patchy pulmonary infiltrates (yellow arrows) concerning for multilobar pneumonia.

A 58-year-old female patient who had a past medical history of essential hypertension, morbid obesity, and surgical history of hysterectomy and cholecystectomy presented to Brookdale University Hospital and Medical Center emergency room (ER) at the time of COVID-19 pandemic peak in New York City with a new onset diffuse left lower extremity (LLE) swelling, numbness, and pain along with back pain for the last 12 hours. Eleven days before presenting to the ER, she called her primary care doctor (PCP) and spoke to him via telemedicine and told her that she started feeling very sick, had headache, fatigue, generalized muscle aches, body temperature of 101°F, sore throat, and cough. She also mentioned that she had lost her sense of smell or taste but denied any chest tightness or shortness of breath. Her physician was concerned about SARS-CoV-2 infection and advised her to go to a clinic and get tested for it. However, she was scared of leaving her house and getting tested. On the initial physical exam in the ER, the patient was awake, alert, and oriented but in mild respiratory distress. Initial vital signs revealed a body temperature of 97.8°F, blood pressure of 149/62 mmHg, heart rate of 75 per minute, respiratory rate of 22 per minute, oxygen saturation of 94% on room air which improved to 100% with supplemental oxygen therapy via non-rebreather mask, and a body mass index of 62 kg/m^2^. She had coarse breath sounds with increased respiratory effort, old midline laparotomy scar and benign abdomen, diffuse swelling and tenderness of her LLE with decreased palpable pulses compared to right lower extremity. There was no discoloration or skin changes of the LLE. Initial chest X-ray (CXR) showed bilateral patchy pulmonary infiltrates concerning multilobar pneumonia that was later demonstrated with CT of the chest (Figure 1).

A presumptive diagnosis of COVID-19 was made, and a nasopharyngeal polymerase chain reaction (PCR) test was done that later confirmed the diagnosis of COVID-19. Initial laboratory investigations are summarized in Table 1, which revealed leukocytosis with neutrophil predominance, mild thrombocytopenia, microcytic anemia with high red cell distribution width (RDW), significantly elevated D-dimer level, acute kidney injury, lactic acidosis, and elevated inflammatory markers most likely secondary to severe sepsis due to COVID-19.

**Table 1:** Initial Laboratory Investigations H: High, L: Low

Component	Value	Reference Range and Units
White blood cells	23.40 (H)	4.10-10.10 × 10^3^/µL
Neutrophils, absolute	16.9 (H)	2.30-6.80 × 10^3^/µL
Lymphocytes, absolute	4.6 (H)	1.30-3.00 × 10^3^/µL
Monocytes, absolute	1.6 (H)	0.30-0.90 × 10^3^/µL
Eosinophils, absolute	0.2	0.00-0.50 × 10^3^/µL
Basophils, absolute	0.1	0.00-0.10 × 10^3^/µL
Hemoglobin	10.8 (L)	11.4-15.5 g/dL
Red cell distribution width (RDW)	16	12.6%-14.9%
Mean corpuscular volume	75.4 (L)	82.0-94.5 fL
Platelets	167 (L)	180-401 × 10^3^/µL
Blood urea nitrogen (BUN)	11	7.0-17.0 mg/dL
Creatinine	1.60 (H)	0.52-1.04 mg/dL
Sodium	139	133-145 mEq/L
Potassium	3.7	3.5-5.1 mEq/L
Chloride	97	98-107 mEq/L
Calcium	8.5	8.4-10.5 mg/dL
Bicarbonate	18.4 (L)	22.0-26.0 mmol/L
Anion gap	28.7	7.00-17.00 mmoL/L
Lactate	11.40 (H)	0.70-2.10 mmol/L
Lactate dehydrogenase (LDH)	1,370 (H)	313-618 IU/L
C-reactive protein (CRP)	27 (H)	0.50-1.00 mg/dL
Ferritin	283 (H)	11.10-264.00 ng/mL
Erythrocyte sedimentation rate (ESR)	30	0-30 mm
International normalized ratio (INR)	1.38 (H)	0.70-1.20
Partial thromboplastin time (PTT)	28.5	23.5-35.5 seconds
D-dimer	52,645 (H)	0-230 ng/mL DDU
Fibrinogen	>1,400	311.0-535.0 mg/dL

**Figure 2:**
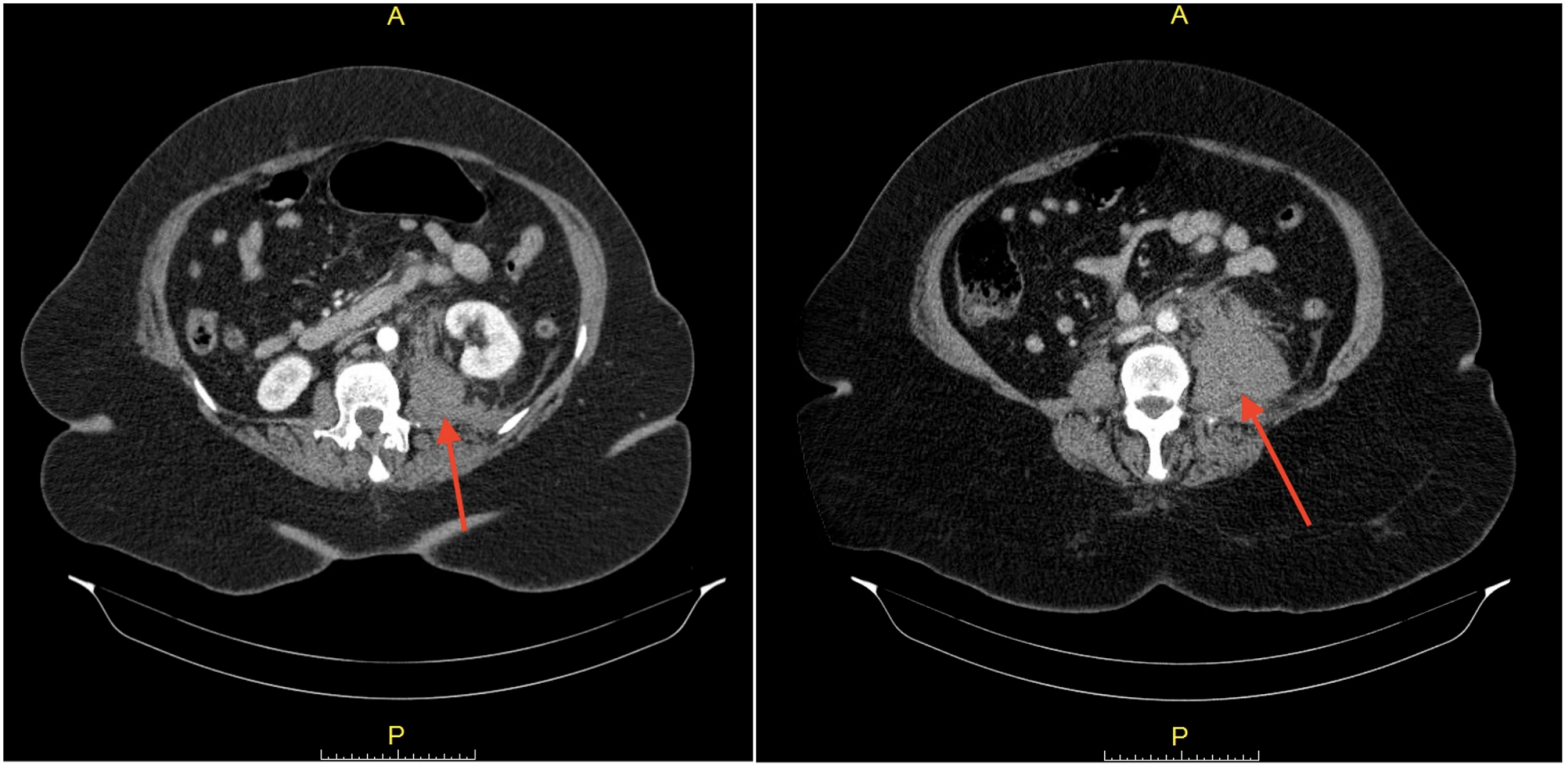
CT angiogram showing a large retroperitoneal hematoma (red arrows) in the left hemipelvis near the origin of the left internal iliac artery and extending along the left iliopsoas muscle and displacing the left kidney anteriorly.

CT angiogram (CTA) of chest, abdomen, and pelvis with contrast was done to rule out pulmonary embolism (PE) in light of hypoxemic respiratory failure and markedly elevated D-dimer, and also to investigate any source of bleeding that might cause an acute drop in hemoglobin, which ruled out PE but showed a large retroperitoneal hematoma in the left hemipelvis (Figure 2).

**Figure 3:**
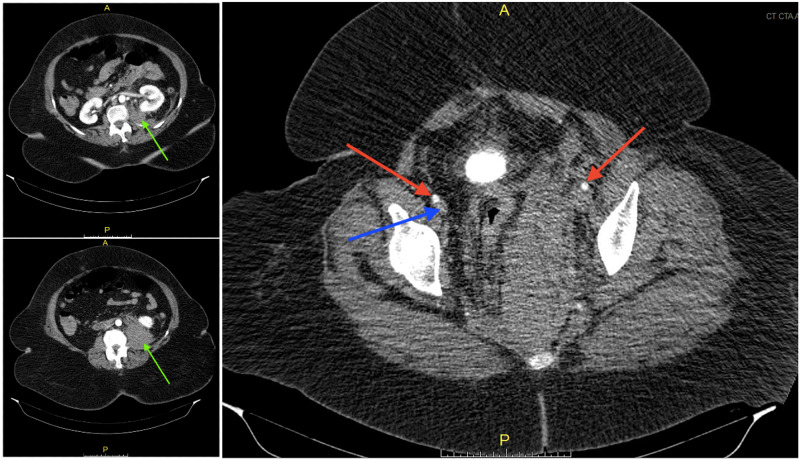
CT angiogram abdominal aorta and bilateral iliofemoral lower extremity runoff with contrast showing a large hematoma (green arrows) extending from the pelvis to the iliopsoas musculature measuring approximately 25 x 10 x 6 cm at the level of the pelvis without extravasation of contrast. The arterial vasculature of the bilateral lower extremities is patent bilaterally (red arrows) but there is greatly impaired venous return at the level of the pelvis with compression of the left common iliac vein by the large hematoma. (Blue arrow is pointing to right femoral vein. Left femoral vein is compressed and not visible.)

Subsequently, venous duplex ultrasound of the LLE was done to rule out deep vein thrombosis (DVT), which showed an acute, non-compressible, occlusive deep venous thrombosis of the left iliofemoral and femoral veins. Further evaluation was done with CTA abdominal aorta with contrast and again showed the large retroperitoneal hematoma without extravasation of the contrast and diffuse swelling of the LLE and compression of the left common iliac vein by the large hematoma (Figure 3).

On the third day of admission, the patient's LLE examination displayed significant edema and pain along with muscle weakness. Compartments were tense but compressible in thigh and legs both. The patient underwent LLE calf and thigh fasciotomy for LLE compartment syndrome with inferior vena cava filtration and transferred to the surgical intensive care unit. The patient experienced significant rhabdomyolysis after fasciotomy with a maximum creatinine phosphokinase (CPK) level of 54,000 U/L which was complicated by acute renal failure and severe hyperkalemia (K level of 6.8 mEq/L) requiring continuous renal replacement therapy (CRRT). The patient had a cardiac arrest for six minutes on postop day 3 and responded to resuscitation. During the remainder of the patient's hospital course, she received convalescent plasma as part of COVID-19 treatment, required vasopressor support for septic shock from COVID-19, and started anticoagulation with enoxaparin and then heparin infusion after experiencing acute renal failure for treatment of DVT and hypercoagulable state. She had a worsening shock despite receiving multiple vasopressors. She was treated for septic shock empirically with meropenem and fluconazole for possible candidemia. Even though she had persistent fever spikes and significantly elevated white blood cell counts with left shift, blood cultures did not grow any organisms but she had Candida albicans in her sputum and urine cultures. Her course was complicated with a second cardiac arrest on postop day 13. Post cardiac arrest laboratory tests showed acute drop in hemoglobin from 7.2 to 4.2 and severe lactic acidosis with a pH of 6.75. Soon after, the patient had a third and fourth cardiac arrest without response to resuscitation efforts and died.

## Discussion

Patients with COVID-19 have a high tendency to develop acute thrombotic events, including VTE, acute stroke, acute myocardial infarction, and clotting of the ECMO (extracorporeal membrane oxygenation) and CRRT catheters [4-9]⁠. Autopsies of patients who died from COVID-19 revealed severe endothelial injury, widespread thrombosis with microangiopathy and alveolar capillary microthrombi, and increased angiogenesis [10]⁠. These findings support the evidence of hypercoagulable state in patients with COVID-19. VTE is commonly seen as a complication of hypercoagulable state in patients with COVID-19. When DVT is severe, it can compromise venous outflow from a limb and can cause significant limb swelling resulting in phlegmasia cerulea dolens (PCD). PCD caused by a massive DVT can further get complicated by compartment syndrome, which is a surgical emergency. Compartment syndrome that develops with PCD is treated with fasciotomy as in our case.

It is quite interesting that our patient presented with spontaneous retroperitoneal bleeding and also massive DVT causing compartment syndrome. To the best of our knowledge, spontaneous bleeding along with massive DVT has not been reported in the current literature, in patients with COVID-19. She did not receive any anticoagulation or had a trauma to the area prior to the CT scan, which makes this case an example of spontaneous bleeding. She also never had any prior history of thrombophilia, she was not on any oral contraceptives, and there were no other obvious factors that might be responsible for development of massive DVT. Our patient initially had borderline low thrombocyte count, normal activated partial thromboplastin time (aPTT, 28.5 seconds) with slightly increased prothrombin time (PT, 16.5 seconds), very high D-dimer (52,645 ng/mL DDU), high fibrinogen (>1,400 mg/dL), and therefore, did not meet the diagnostic criteria for acute DIC.

Even though patients who have severe COVID-19 have a tendency to develop thrombotic complications, they also can less frequently have bleeding complications. A multicenter study reported the hemostatic manifestations, bleeding, and thrombotic complications of 400 COVID-19 patients [11]⁠. It showed a radiographically confirmed venous thromboembolic rate of 4.8% (7.6% in critically ill patients) with an overall thrombotic complication rate of 9.5%. In contrast, the overall bleeding rate was 4.8% (7.6% in the critically ill patients), with a major bleeding rate of 2.3% (5.6% in the critically ill, including one fatal bleed). A meager rate of DIC was noted in 2% of the critically ill patients. D-dimer levels were increased far out of proportion to any abnormalities in the PT/INR (international normalized ratio), aPTT, fibrinogen level, or platelet count; these findings are uncharacteristic of DIC as currently understood. Therefore, coagulopathy in COVID-19 is significantly different from severe sepsis from other causes. The remarkable combination of thrombocytopenia, prolonged PT, and elevated D-dimer can suggest DIC, although it is different from DIC seen in sepsis. In sepsis, thrombocytopenia is more profound, and D-dimer concentrations do not reach the high values observed in patients with COVID-19. According to the DIC score of the International Society on Thrombosis and Haemostasis, most patients with COVID-19 would not be classified as DIC. COVID-19 post-mortem findings show typical microvascular platelet-rich thrombotic depositions in small vessels of the lungs and other organs. However, there are no signs of hemolysis or schistocytes in the blood film, and the platelet count is higher than would be expected in case of thrombotic microangiopathy. It is suggestive that coagulopathy associated with COVID-19 is a combination of low-grade DIC and localized pulmonary thrombotic microangiopathy [12]⁠.

## Conclusions

Patients with severe COVID-19 have a high tendency to develop hypercoagulable state and its acute thrombotic complications affecting different organ systems in critically ill patients. Even though thrombotic events are frequently reported in patients with COVID-19, incidence of bleeding is far less common and it is usually associated with antiplatelet agents and anticoagulation. In addition, COVID-19 coagulopathy is quite different from DIC caused by severe sepsis of other causes. To the best of our knowledge, massive thrombosis along with spontaneous bleeding in patients with COVID-19 has not been reported. We hope that this case report will contribute to our understanding of COVID-19 related coagulopathy.
